# Adult duck fecal microbiota transplantation alleviates short beak and dwarfism syndrome in ducklings by inhibiting Th17 cell differentiation

**DOI:** 10.1080/21505594.2025.2605745

**Published:** 2025-12-16

**Authors:** Mandi Liu, Weining Liu, Kuan Zhao, Wuchao Zhang, Baishi Lei, Yunhang Zhang, Limin Li, Wanzhe Yuan

**Affiliations:** a College of Veterinary Medicine, Hebei Agricultural University, Baoding, China; b National Research Center of Engineering and Technology for Veterinary Biologicals, Nanjing, China; c College of Animal Science and Technology, Chongqing Three Gorges Vocational College, Chongqing, China

**Keywords:** NGPV, SBDS, FMT, intestinal microbiota, Th17 cell

## Abstract

Novel goose parvovirus (NGPV) infection in ducklings induces short beak and dwarfism syndrome (SBDS), leading to significant economic losses. Since NGPV predominantly infects ducklings, whether reshaping the intestinal flora of ducklings through fecal microbiota transplantation from adult ducks (FMT-A) can alleviate SBDS is an interesting question. This study aimed to investigate the impact of FMT-A on the susceptibility of ducklings to NGPV infection, to elucidate the potential relationship between gut microbiota and viral pathogenicity. The results showed that ducklings were more susceptible to NGPV than adults, and that adult ducks exhibited higher fecal microbiota richness and diversity. FMT-A treatment attenuated NGPV-induced reductions in body weight, beak and tibia length, and muscle mass. Furthermore, FMT-A alleviated gut dysbiosis and intestinal tissue damage, increased glycogen in the intestinal mucosa, upregulated ZO-1 expression, expanded the epiphyseal region, and reduced osteoclast numbers in the tibia of ducklings. Moreover, FMT-A suppressed the expression of the Th17 cell-specific transcription factor retinoic acid receptor-related orphan receptor γt in the ileum and bone, and decreased the expression levels of pro-inflammatory cytokines in the ileum, bone, and serum. These findings indicate that ducklings are more susceptible to NGPV than adult ducks, with significantly lower diversity and abundance of fecal microbiota. FMT-A can stabilize intestinal flora, mitigate intestinal barrier damage, inhibit Th17 cell differentiation, thereby reducing abnormal bone development, and ultimately alleviate SBDS in ducklings. These findings provide a theoretical basis for developing novel strategies targeting gut microbiota modulation to prevent and control SBDS in ducklings.

## GRAPHICAL ABSTRACT

**Figure:**
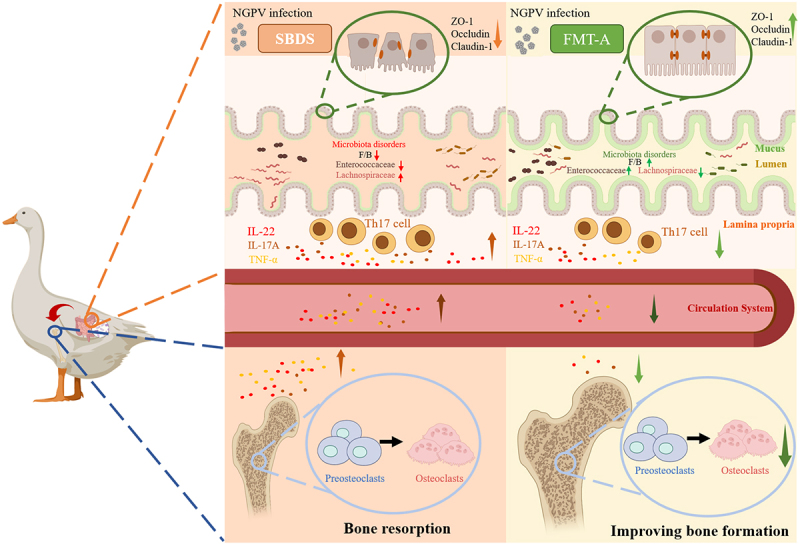

## Introduction

Novel goose parvovirus (NGPV) primarily infects Cherry Valley ducks, causing short beak and dwarfism syndrome (SBDS), with clinical manifestations including shortened beaks, exposed tongues, dwarfism, and a predisposition to fractures [1]. Since ducks exhibit persistently slow growth after recovery, the qualification rate decreases, causing significant economic losses in the breeding industry [2]. The gut microbiota plays a crucial role in maintaining intestinal immune homeostasis. A balanced gut microbiota can effectively prevent intestinal inflammation [3,4], whereas dysbiosis may exacerbate intestinal inflammatory conditions [5]. In recent years, fecal microbial transplantation (FMT) technology has garnered increasing attention from researchers as it has emerged as an effective method for reconstructing dysregulated intestinal microbiota. For instance, studies have demonstrated that FMT can significantly enhance the growth performance of broilers [6]. Moreover, the transplantation of fecal microbiota from 208-day-old chickens into newly hatched chicks has been shown to mitigate the adverse effects associated with *Salmonella* infection and decrease the mortality rate in chicks [7]. Therefore, transplanting the microbial flora from adult animals to newborn animals may be a potential strategy for reducing pathogen infections. Since our previous research has confirmed that the intestinal microbiota of ducklings participates in the pathogenesis of SBDS [8], however, there has been no report indicating whether there are significant differences in the microbial flora between the feces of adult and duckling in Cherry Valley ducks; And whether transplanting the microbial flora from adult ducks into the ducklings can promote the growth and development of the ducklings as it did in the broilers remains unknown [6]. Therefore, comparing the differences in microbial flora between ducklings and adult ducks and studying whether transplanting the microbial flora from adult ducks to ducklings can improve the growth performance of SBDS ducklings is an interesting question.

There are various immune cells in the intestinal tract, such as Th cells and Treg cells, which collaborate to maintain the balance of the immune response; otherwise, intestinal inflammation may occur. The pro-inflammatory cytokines, mainly produced by intestinal immune cells, including interleukin (IL) 6, IL-1β, are accompanied by a less amount of anti-inflammatory cytokines, such as IL-10 and TGF-β. Pro-inflammatory cytokines are mainly produced by Th cells, especially Th17 cells, while anti-inflammatory cytokines are mainly produced by Treg cells [9,10]. It has been established that chronic intestinal inflammation is closely associated with the differentiated Th17 through the critical transcriptional factor retinoic acid receptor-related orphan receptor γt (ROR γt) and the key cytokines such as IL-17A, IL-22, and tumor necrosis factor-α (TNF-α) [11]. The chronic inflammatory response triggered by the activation of Th17 cells not only leads to the production of cytokines and the recruitment of neutrophils and other inflammatory cells but is also closely associated with the intestinal microbiota. Studies have demonstrated that Th17 cells have a microbiota-dependent role in the arthritis mouse model [12]. Additionally, studies have demonstrated that the imbalance of Th17/Treg cells results in reduced growth performance in chickens, whereas gut microbiota reshaped by FMT can enhance growth performance by restoring the balance of Th17/Treg cells [11]. Therefore, modulating the intestinal flora to alleviate Th17 cell differentiation represents a feasible approach to improving the growth performance of SBDS ducklings.

The differentiation of Th17 cells not only leads to the release of pro-inflammatory factors, thereby causing intestinal inflammation, but also directly or indirectly modulates the receptor activator of NF-κB ligand (RANKL)/receptor activator of NF-κB (RANK)/osteoprotegerin (OPG) pathway [13]. RANKL binds to RANK on the surface of osteoclasts and their precursor cells, promoting the differentiation of osteoclast precursors and facilitating the formation of osteoclasts. Chen [14] demonstrated that bone loss and bone remodeling are related to the ratio of RANKL to OPG expression, and that the expression level of RANKL is positively correlated with the number of Th17 cells. IL-17 secreted by Th17 cells can indirectly stimulate osteoclastogenesis and promote bone resorption [15]. Although our previous research has shown that NGPV infection can lead to intestinal inflammation and bone loss in SBDS ducklings [16]. The influence of NGPV infection on intestinal immune cells, especially Th17 cell differentiation, has not been documented. It remains unclear whether modulating the intestinal microbiota can alter the intestinal immune status and bone metabolic status induced by NGPV infection. Therefore, this study aims to utilize FMT to determine whether the colonization of fecal microbiota from adult ducks can regulate the balance of Th17 cells and alleviate the bone metabolic imbalance in NGPV-infected ducklings, providing new insights for the prevention and treatment of SBDS in ducklings.

## Materials and methods

### Viruses and animals

Strain SD of NGPV was previously isolated and maintained in our lab at −80 °C, and was used as the challenge virus in this study. The complete genome sequence of strain SD has been deposited in GenBank (accession number KU516831). The virus was obtained through passage and amplification in specific pathogen-free (SPF) duck embryos and subsequently stored at −80 °C. The SPF duck embryos were purchased from Shandong Haotai Laboratory Animal Breeding Co., Ltd., and the 1-day-old unvaccinated male Cherry Valley ducklings and 40–50-day-old male fattening ducks were purchased from Anxin Wendong farm located in Baoding, Hebei.

The study utilized animals in compliance with the Laboratory Animal Guideline for Ethical Review of Animal Welfare in China (GB/T 42,011–2022) and received approval from the Animal Welfare and Ethics Committee at the Laboratory Animal Center of Hebei Agricultural University (2024010).

The duration of experimental implementation and data collection for this research spanned from 1 October 2024, to 23 January 2025.

### Experimental method for assessing the susceptibility of Cherry Valley ducks to the NGPV

Our study followed the ARRIVE guidelines (https://www.nc3rs.org.uk/arrive-guidelines). Twenty adult ducks were randomly assigned to 2 groups (*n* = 10/group), the control and the infected groups. Both groups were housed in isolators under controlled temperature and humidity conditions, with free access to water and feed. Following a 3-day adaptation period, the ducks in the infected group were intramuscularly injected with 0.4 mL of NGPV allantoic fluid and orally gavaged with 1 mL of NGPV allantoic fluid (total infection dose: 10^5.25^ EID_50_). The dosage was adjusted based on the experience accumulated in our previous studies [8]. As our previous research found that the lethality rate of this virus was not high, in order to ensure the successful establishment of the model in 6-day-old ducklings, the virus challenge dose was appropriately increased in this study. whereas the ducks in the control group received an equivalent volume of PBS. Next, body weight, beak length, and width, and tibia length were measured weekly for 3 weeks. Anal swabs were collected to detect viral load, and fecal samples were gathered for 16S rRNA sequencing analysis at 21 days post-infection (dpi). The duration of this phase of the study was 25 days in total.

The rearing conditions, grouping, and NGPV infection methods for 1-day-old Cherry Valley ducklings were identical to those used for adult ducks. Feces were collected at 21 dpi for 16S rRNA sequencing, and continuous observation was carried out until the end of the experiment at 28 dpi. The duration of this phase of the study was 32 days in total.

### Modeling method of feeding the feces microorganisms of adult ducks to ducklings

**Figure 1.**
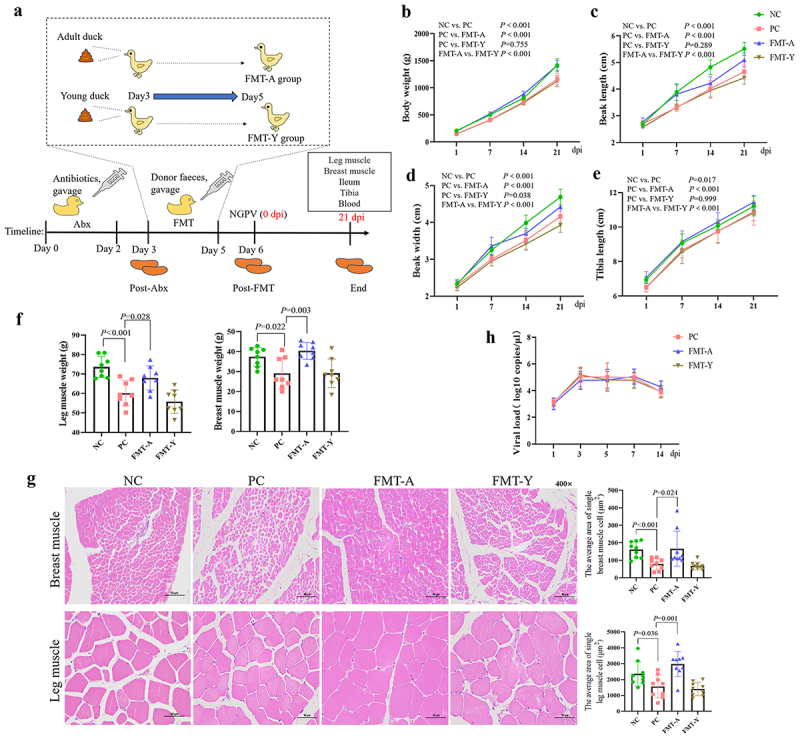
The impact of fecal microbiota transplantation from adult ducks on the symptoms of SBDS in ducklings infected with NGPV. (a): Experimental design: first, Abx were gavaged to ducklings for 3 days. Then the feces collected were labeled as Post-Abx. Next, the feces from untreated healthy adult ducks were gavage transplanted into the Abx-treated ducklings, as the FMT-A group (*n* = 12), while the feces from the untreated healthy ducklings were gavage transplanted into the Abx-treated ducklings, as the FMT-Y group (*n* = 12). Both gavage administrations lasted for 3 days. On Day 6, the feces of the ducklings were collected again and labeled as Post-FMT, followed by the NGPV infection, and the observations continued for 21 days. The leg muscles, breast muscles, ileum, tibia, and blood of the ducklings were collected at 21 dpi. The comparison of body weight (b), Beak length (c), Beak width (d), and tibia length (e) changes in the 4 groups. (f): Compare the weights of leg muscle and breast muscle among the 4 groups. (g): Comparison of the average area of single breast muscle cells and leg muscle cells among the 4 groups (HE staining). (h): Compare the viral load of NGPV in anal swabs from 3 virus-infected groups by qPCR.

The duckling modeling design is presented in Figure 1(a). Forty-eight 1-day-old ducklings were randomly assigned to 4 groups (*n* = 12/group): NC (no infected), PC (Infected only), FMT-A (fecal microbiota transplantation from adult ducks), and FMT-Y (fecal microbiota transplantation from young ducks). Ducklings in the FMT-A and FMT-Y groups received antibiotic treatment for 3 days to deplete their intestinal microbiota. Subsequently, they were gavaged daily with fecal microbiota from adult ducks or ducklings for 3 consecutive days to reconstitute the intestinal microbiota. On the 6th day, PC, FMT-A, and FMT-Y groups were infected with NGPV (total infection dose: 10^5.25^ EID_50_). Observation of the experiment was continued until 21 dpi, then, at least 8 ducks from each group were euthanized by cervical dislocation by professional staff, and tissue samples were collected for further analysis. The duration of this phase of the study was 28 days in total.

### Gut microbiota consumption

The intestinal microbiota depletion model was designed with reference to the methods of Winkler, Thackray LB, and Yang [17–19], and was appropriately adjusted. In the drinking water of ducklings, ampicillin (Solarbio, Beijing, Cat: A6920), neomycin sulfate (Solarbio, Beijing, Cat: IN01302), and metronidazole (Solarbio, Beijing, Cat: M8060) were added at a final concentration of 1 g/L for 3 days. Concurrently, gavage administration was performed, with ampicillin (200 mg/kg), neomycin sulfate (200 mg/kg), metronidazole (200 mg/kg), and vancomycin hydrochloride (40 mg/kg) (Solarbio, Beijing, Cat: V8050) administered once daily for 3 days.

### Gut microbiota reconstruction

The establishment of the gut microbiota reconstruction model also referred to the above-mentioned literature [17–19] and made appropriate adjustments. To identify optimal FMT donors, we selected 5 healthy adult ducks with higher body weights. 5 g of fresh fecal samples were collected from adult ducks and healthy ducklings separately. mixed with 10 mL of physiological saline and sterile steel beads in separate tubes, and homogenized at 45 Hz for 1 min, the homogenates were then filtered through a 70 μm filter to obtain the filtrate. Ducks in the FMT treatment group were administered 0.5 mL of the filtrate per duck daily via gavage once a day for 3 days.

### Verify the success of gut microbiota consumption/transplantation

According to the duckling modeling design in Figure 1(a), at post-Abx and post-FMT, collect duck anal swabs (*n* = 3/group) and immerse them in 0.5 mL of sterile physiological saline. After 30 seconds of vortexing, take 100 μL of the suspension and spread it on a 5% sheep blood agar plate. Place the plate in an anaerobic drying jar at 37 °C for 48 h, followed by incubation in an aerobic environment at 37 °C for 24 h. Observation of the growth status of the bacterial colonies on the plate [19].

### Samples collection

The body weight, beak length, beak width, and tibia length of ducklings were measured weekly after NGPV infection. Anal swabs were collected weekly to detect the viral load of NGPV. The ducks were euthanasia after 12 h of fasting, the weights of the leg and breast muscles were determined, the complete tibia tissue was dissected, and the length and width were measured. For analysis of gut microbiota, ileal contents were collected into sterile 1.5 mL centrifuge tubes and subsequently stored at −80 °C for sequencing. For histological examination, fresh muscle, ileum, and proximal tibia tissues were fixed in 4 % paraformaldehyde solution. Additionally, for gene expression analysis, terminal ileum and proximal tibia tissues were dissected, immediately frozen in −80 °C.

### Detection of NGPV viral load

The duck anal swabs were suspended in 0.5 mL of sterile normal saline, vortexed, and mixed thoroughly. Subsequently, DNA was extracted from the anal swabs using a DNA extraction kit (TransGen Biotech, Beijing, Cat: EE101-12). Using the extracted DNA as the template, Quantitative real‑time polymerase chain reaction (qPCR) was performed in a total reaction volume of 20 μL, which included 1 μL of template DNA, 1 μL of each primer, 10 μL of SYBR Green Master Mix (Vazyme, Nanjing, Cat: Q711-03), and 7 μL of ddH_2_O. The qPCR reaction followed a two-step protocol: initial denaturation at 95 °C for 5 min, followed by 40 cycles of 95 °C for 10 s and 56 °C for 30 s, with a final hold at 16 °C. Primer sequences used for NGPV detection are listed in Table 1.

**Table 1.** Primer design.

Name	Sequence (5”−3”)	Tm (°C)	Product Size (bp)
RORγt	Forward: CTCTTCAGGTCCCTGGGCTG	56	181
	Reverse: GAAAAGCTCCCGGTAGAGCG		
IL-17A	Forward: ACCCTTCGTGCTTCTCTGTC	56	110
	Reverse: GCATCTTTTTGGGTCAGGCA		
IL-22	Forward: TTCCTGGCATCCCTGACCTC	56	122
	Reverse: ATTCTTTCCATTCTCTCCCAACTGT		
TNF-α	Forward: CCGCCCAGTTCAGATGAGTTGC	56	97
	Reverse: GCCACCACACGACAGCCAAG		
RANKL	Forward: TAAGTTTGCCTGGCCTTTGT	56	100
	Reverse: GCCTTTTGCCCATCTCATTA		
OPG	Forward: GAAGGTCTGCTCTTGCGAAC	56	106
	Reverse: GCCTAACTGGCTGAACTTGC		
GAPDH	Forward: GCTTTCCCGTGTGCCAACCC	56	116
	Reverse: GCCCATCAGCAGCAGCCTTC		

### Histopathological staining

Hematoxylin and eosin (HE) staining. The leg muscle, breast muscle, ileum, and tibia tissues were processed for paraffin embedding and subsequently sectioned into 5 μm slices. The tissue sections were subjected to HE staining, following the manufacturer’s instructions provided in the H&E staining kit (Solarbio, Beijing, Cat: G1100).

Periodic acid-Schiff (PAS) staining. Following the preparation of paraffin-embedded tissue sections, dewaxing and rehydration were performed. The sections were then oxidized with periodic acid solution for 10 min, followed by washing with distilled water. Schiff reagent was applied for 10 min, and excess reagent was removed by rinsing under running water. After drying, the sections were counterstained with hematoxylin, mounted with neutral resin, and examined under a microscope, with photographs taken.

Immunohistochemistry (IHC). After dewaxing and rehydration of the sections, endogenous peroxidase activity was blocked with 0.3 % H_2_O_2_. Antigens were retrieved using EDTA antigen retrieval solution (Boster, Wuhan, Cat: AR0023). Antigen blocking was performed with 3% bovine serum albumin in a wet box at 37 °C for 30 min. The primary antibody: ZO-1 (Cat: A0659) and RORγt (Cat: A10240) (ABclonal, Wuhan) was applied to the tissue and incubated at 37 °C for 2 h. After washing with PBS, an HRP-labeled secondary antibody (ABclonal, Wuhan, Cat: AS014) was added and incubated at 37 °C for 30 min. Following another PBS wash, the diaminobenzidine chromogenic solution (Solarbio, Beijing, Cat: DA1016) was prepared according to the manufacturer’s instructions and applied to the tissue. Finally, tissues were counterstained with hematoxylin and observed under a microscope.

Tartrate-resistant acid phosphatase (TRAP) staining. Tibias were fixed in 4 % paraformaldehyde for one week, followed by decalcification in 12.5 % EDTA for 12 weeks. The tissues were then dehydrated through an ethanol gradient and embedded in paraffin; the paraffin-embedded blocks were sectioned at a thickness of 5 μm. Tartrate-resistant acid phosphatase (TRAP) staining kit (Solarbio, Beijing, Cat: G1495) was used to stain osteoclasts in the tissues. Microscopic observations and photographs were taken, and the number of osteoclasts per bone surface (N.Oc/BS) was quantified.

### Detection of cytokines

The concentrations of serum cytokines IL-17A (Cat: BY-ED662658), IL-22 (Cat: BY-ED662651), and TNF-α (Cat: BY-ED661032) were measured using enzyme-linked immunosorbent assay (ELISA) kits (BYabscience, Nanjing).

### Quantitative real‑time polymerase chain reaction (qPCR)

After grinding and homogenizing the tissues, total RNA was extracted from the ileum and tibia using TRIzol (Takara, Beijing, Cat: 9109). Subsequently, RNA was reverse transcribed into cDNA using a reverse transcription kit (Vazyme, Nanjing, Cat: R223-01). The primer sequences for the target genes are listed in Table 1. qPCR was performed using cDNA as the template to analyze the expression levels of the target gene. Glyceraldehyde-3-phosphate dehydrogenase (GAPDH) was used as the housekeeping gene. 3 technical replicates were set for each sample, and the average Ct value of these replicates was utilized for data analysis. The relative expression levels of the target genes were calculated using the 2^−ΔΔCt^ method.

### 16S rRNA gene sequencing and analysis

The bacterial 16S rRNA gene sequencing of duck feces and ileal contents was conducted by Novogene Co., Ltd. Small fragment libraries were constructed based on the characteristics of the amplified regions, followed by paired-end sequencing using the Illumina NovaSeq platform. After Reads splicing and filtering, operational taxonomic units (OTUs) clustering or amplicon sequence variants (ASVs) denoising, the resulting valid data were subjected to species annotation and abundance analysis to reveal the species composition of the samples.

Raw sequencing data underwent splicing and filtering to produce valid data (Clean Data). Afterward, noise reduction was applied to the Clean Data using DADA2 [20], which yielded the final ASVs. For the ASVs obtained [21], species annotation was performed on the representative sequences of each ASV, allowing for the determination of species information and abundance distribution at the species level. Concurrently, analyses such as abundance profiling and alpha diversity calculations were conducted to identify common and unique ASVs across different groups. Principal Coordinates Analysis (PCoA) was utilized for multiple sequence alignments of ASVs, facilitating the assessment of community structure differences among groups. To further explore variations in community structure among the grouped samples, the LEfSe statistical method was employed to perform significance testing on species composition and community structure.

### Statistical analysis

The average cross-sectional area of muscle cells and the number of osteoclasts were quantified using ImageJ-1.51J8, and the length of ileal villi and the depth of crypts were also measured. 3 tissues were selected for each group, and three random fields of view were selected for each tissue for measurement. All data were graphically represented and statistically analyzed using Prism 9.0.0. The data are presented as the means ± SD. Statistical analysis of the results was conducted using one-way ANOVA and Independent Sample t-test; the value (*p* < 0.05) was considered statistically significant.

## Results

### Adult ducks are more susceptible to NGPV infection than young ducks

**Figure 2.**
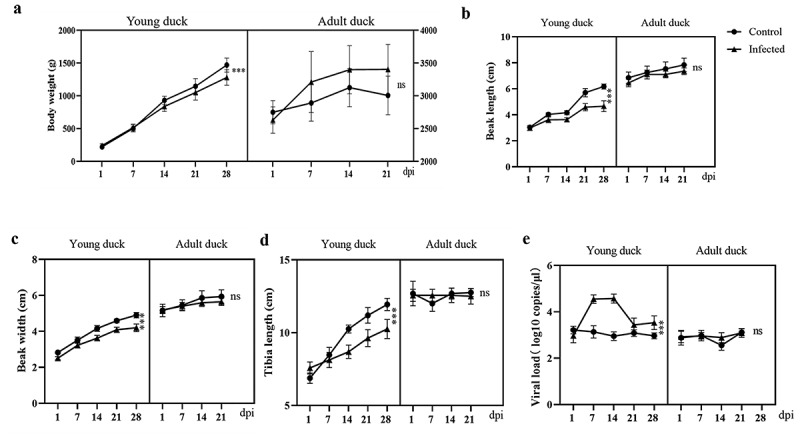
Comparison of susceptibility to NGPV between ducklings and adult ducks. the body weight (a), Beak length (b), Beak width (c), and tibia length (d) Changes of young ducks and adult ducks after NGPV infection. (e): Viral loads in anal swabs of ducklings and adult ducks after NGPV infection. Data are shown as mean ± SD. ***p < 0.001; ns, *p* > 0.05.

In young ducks, NGPV infection resulted in significant reductions in body weight (Figure 2(a)), beak length (Figure 2(b)), beak width (Figure 2(c)), and tibia length (Figure 2(d)). In contrast, these indicators remained unaffected in adult ducks following NGPV infection. Furthermore, the viral load of NGPV in anal swabs from ducklings peaked at 7 and 14 dpi and was significantly higher than that in the control group (Figure 2(e)). However, no substantial changes in viral load were observed in adult ducks. The above results indicate that ducklings are more susceptible to NGPV than adult ducks.

### The microbial composition of feces in healthy adult ducks and young ducks is different

**Figure 3.**
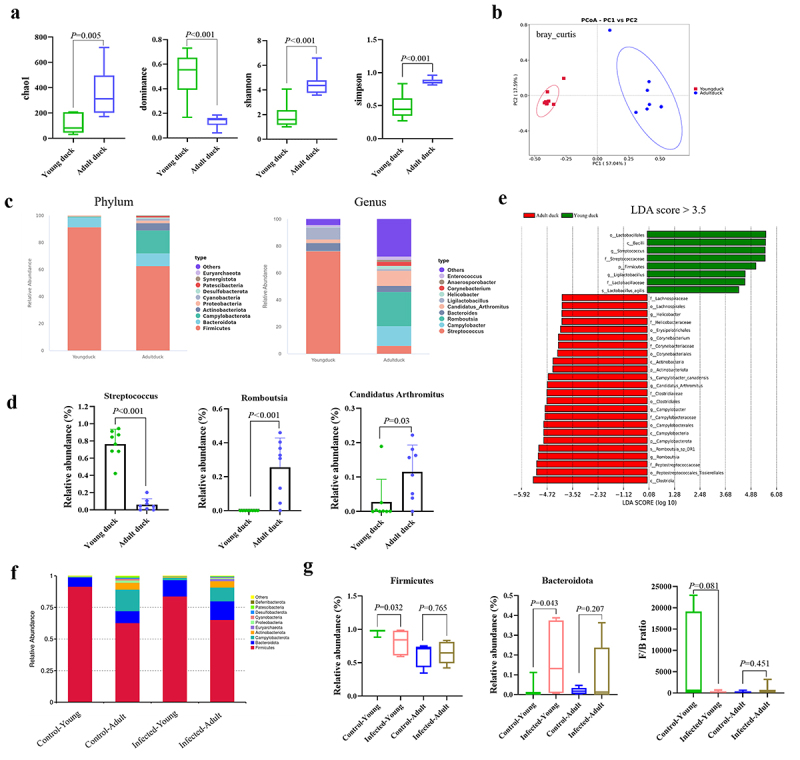
Comparison of the differences in the composition of fecal microorganisms between young ducks and adult ducks. (a): The comparison of the microbial α-diversity in the feces of healthy ducklings and adult ducks, including the Chao1, dominance, Shannon, and Simpson indices. (b): PcoA was used to compare the β-diversity of fecal microorganisms in healthy young ducks and adult ducks. (c): Bar charts representing the relative species abundance of bacteria in the feces of healthy young ducks and adult ducks at both the phylum and genus levels. (d): Compare the differences in relative abundances of *Streptococcus*, *Romboutsia*, and *candidatus arthromitus* in the feces of healthy young ducks and adult ducks. (e): Through LEfSe analysis, differential species with an LDA score > 3.5 were identified to compare the differences in fecal microbiota composition between healthy young ducks and adult ducks. (f): The column chart illustrates the phylum-level relative abundance of fecal microorganisms in young ducks and adult ducks at 21 days post-NGPV infection. (g): Compare the effects of NGPV infection on the relative abundances of *Firmicutes* and *Bacteroidota*, as well as the F/B ratio in the feces of young ducks and adult ducks.

To analyze whether the pathogenicity of NGPV is associated with the intestinal microbiota, we collected fecal samples from healthy ducklings and adult ducks and analyzed the compositional differences of fecal microorganisms using 16S rRNA gene sequencing. The α-diversity indices, including Chao1, Dominance, Shannon, and Simpson indices (Figure 3(a)), revealed that the Chao1, Shannon, and Simpson indices in adult ducks were significantly higher than those in the ducklings, whereas the Dominance index was lower in adult ducks compared to ducklings. These findings suggest that the fecal microbiota of adult ducks exhibits greater species richness and diversity than that of ducklings. The β-diversity of fecal microorganisms was analyzed using PCoA (Figure 3(b)). The results demonstrated that the sample distribution of ducklings was significantly distinct from that of adult ducks, indicating substantial differences in intestinal microbial community structure between adult ducks and ducklings.

Analysis of the relative abundance distribution of intestinal microorganisms in the feces of ducklings and adult ducks is shown in Figure 3(c). At the phylum level, *Firmicutes* constituted a significantly larger proportion in both duckling and adult duck feces, followed by *Bacteroidota*. However, the microbial community in the feces of adult ducks exhibited greater diversity compared to that of ducklings. At the genus level, the relative abundance of *Streptococcus* in the feces of adult ducks decreased markedly compared with ducklings, whereas the relative abundances of *Romboutsia* and *Candidatus Arthromitus* increased significantly (Figure 3(d)). The distribution of differential strains was analyzed using LEfSe, and with the LDA score >3.5. The results indicated that the differential strains were predominantly found in adult ducks, including taxa such as *Clostridia*, *Peptostreptococcales_Tissierellales*, and *Romboutsia*. In contrast, *Lactobacillales*, *Bacilli*, and *Streptococcus*, as the main differential bacteria, were enriched in the feces of ducklings (Figure 3(e)).

Next, we observed the effects of NGPV virus infection on the fecal microbiota of ducklings and adult ducks (Figure 3(f)). The results demonstrated that compared with Control ducklings, NGPV infection caused a significant reduction in the abundance of *Firmicutes* and a notable increase in the abundance of *Bacteroidota* in infected young ducks, leading to a decrease in the F/B ratio. In contrast, these changes were not observed in the fecal microbiota of adult ducks (Figure 3(g)). In conclusion, significant differences exist in the microbial composition of feces between adult ducks and ducklings, and NGPV infection exerts a relatively limited influence on the fecal microbiota in adult ducks.

### Adult duck fecal microbiota transplantation mitigates SBDS and enhances the growth performance of ducklings

The experimental animal models were established as shown in Figure 1(a). To verify the success of the intestinal microbial depletion model induced by antibiotics (Abx) treatment and the intestinal microbial reconstruction model via FMT. Fresh fecal samples were collected and inoculated onto blood agar media. The results are shown in Fig. S1a. Scattered pinpoint single colonies were sporadically observed on the blood plates inoculated with Post-Abx duck feces, whereas confluent bacterial lawns grew on the blood plates inoculated with NC, Post-FMT-Y, and Post-FMT-A duck feces. These findings confirm that Abx treatment effectively depleted the intestinal microbiota, and FMT successfully restored the intestinal flora. Furthermore, the ducklings infected with NGPV exhibited shorter beaks and protruding tongues, which are typical symptoms of SBDS, indicating that our NGPV infection model was also successfully constructed (Fig. S1b). Subsequently, 16S rRNA sequencing was conducted on the fecal samples of Post-FMT-A and Post-FMT-Y. The PCoA results revealed that the distribution of Post-FMT-A samples was significantly separated from that of Post-FMT-Y samples (Fig. S1c), indicating substantial differences in the microbial structure of duck feces between these two groups. Furthermore, compared with the FMT-Y group, the FMT-A group exhibited a decreased abundance of *Firmicutes* (Fig. S1d), an increased abundance of *Bacteroidota*, and a reduced Firmicutes-to-Bacteroidota (F/B) ratio (Fig. S1e). These results align with previous observations, suggest that FMT technology can effectively alter the composition of fecal microorganisms in ducklings, bringing it closer to the microbial profile observed in adult ducks.

The body weight (Figure 1(b)), beak length (Figure 1(c)), beak width (Figure 1(d)), and tibia length (Figure 1(e)) were measured weekly; the results showed that these indicators in the FMT-A group were significantly higher than those in the PC group and the FMT-Y group. This suggests that fecal microbiota transplantation from adult ducks can alleviate SBDS in ducklings. Additionally, at 21 dpi, FMT-A treatment significantly increased the body weight, breast muscle weight, and leg muscle weight of ducklings, whereas FMT-Y treatment showed no significant difference compared to the PC group (Figure 1(f)). Histological analysis via HE staining of breast and leg muscles revealed that NGPV infection significantly reduced the average area of single muscle cells, while FMT-A treatment restored that in ducklings (Figure 1(g)). These findings indicate that FMT from adult ducks can mitigate growth and development disorders induced by NGPV infection. Furthermore, the NGPV viral load in duck anal swabs (Figure 1(h)) showed no significant differences among the PC, FMT-A, and FMT-Y groups, suggesting that changes in intestinal microbiota do not influence NGPV excretion.

### Adult duck fecal microbiota transplantation sustains the homeostasis of the ileal microbiota in NGPV-infected ducklings

**Figure 4.**
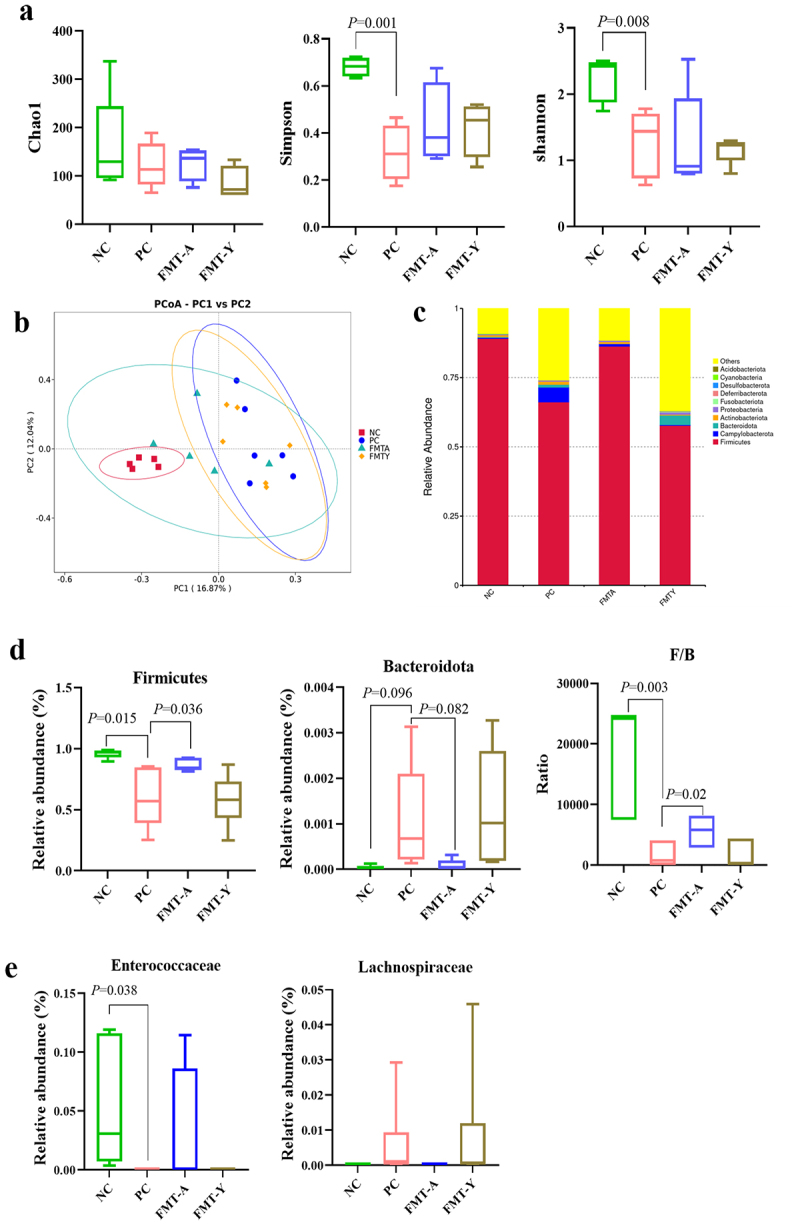
The impact of fecal microbiota transplantation from adult ducks on the ileal microbiota composition in NGPV-infected ducklings. (a): Compare the α diversity of microorganisms in the four groups. (b): Compare the β diversity of microorganisms in the four groups by PCoA. (c): The column chart of the relative abundance of the ileum bacteria in 4 groups at the phylum level. (d): Compare the relative abundances of *Firmicutes* and *Bacteroidota* and the F/B ratio in each group. (e): Compare the relative abundances of *enterococcaceae* and *lachnospiraceae* in each group.

The differences in the ileal microbiota at 21 dpi of NGPV infection were compared by using 16S rRNA gene sequencing. The results demonstrated that NGPV infection significantly reduced the α-diversity of the ileal microbiota, as reflected by decreases in the Chao1, Simpson, and Shannon indices. While FMT-A treatment tended to restore microbial diversity, the improvement did not reach statistical significance (Figure 4(a)). The PCoA analysis results of microbial β diversity revealed that the distributions of PC and NC samples were significantly separated. Notably, the FMT-A group samples exhibited a distribution pattern closer to the NC group and distinctly separate from both the PC and FMT-A groups (Figure 4(b)). Next, the relative abundances of bacteria at the phylum level in each group were analyzed (Figure 4(c)). The results indicated that FMT-A treatment effectively mitigated the significant decrease in the relative abundance of *Firmicutes* and the increase in the relative abundance of *Bacteroidota* (*p* > 0.05) induced by NGPV infection, as well as the marked reduction in the F/B ratio (Figure 4(d)). Moreover, NGPV infection resulted in a significant decline in *Enterococcaceae* relative abundance and an elevation in the level of *Lachnospiraceae* in the ileum of ducklings in both the PC group and the FMT-Y group. However, ducks treated with FMT-A exhibited profiles similar to those of the NC group (Figure 4(e)). These findings suggest that NGPV infection induced substantial alterations in the intestinal microbial structure of ducklings, whereas FMT-A treatment partially restored the intestinal microbial balance in affected ducks.

### Adult duck fecal microbiota transplantation mitigates intestinal barrier dysfunction in SBDS ducklings

**Figure 5.**
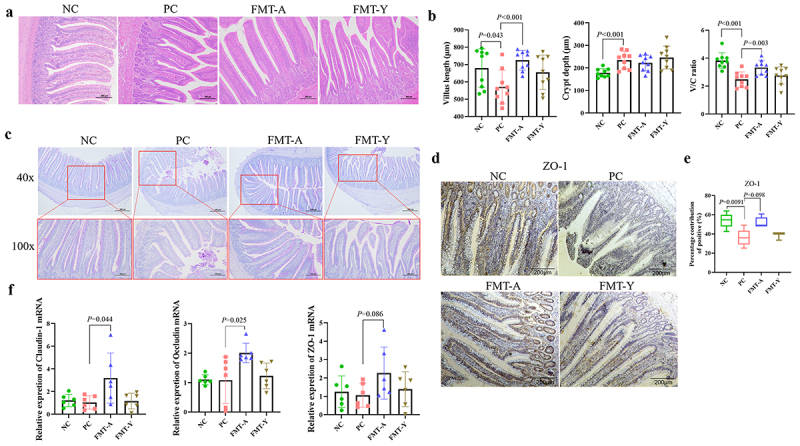
The impact of fecal microbiota transplantation from adult ducks on the intestinal barrier. (a): Comparison of HE staining of the ileum tissues in each group. (b): Compare the ileal villus length, crypt depth, and the villus-to-crypt (V/C) ratio among the groups. (c): Compare the ileal mucus layers of each group by PAS staining. (d): Compare the protein expression levels of ZO-1 in the ileum of each group using immunohistochemical staining. (e): The contribution of positive area in immunohistochemistry. (f) The relative mRNA expression levels of tight junction proteins, including Claudin-1, Occludin, and ZO-1, were compared across the 4 groups by qPCR.

The intestinal microbiota can affect the homeostasis of the intestinal barrier. Thereby, in this study, the ileal structure of ducklings was examined using HE staining (Figure 5(a)). The results demonstrated that FMT-A treatment effectively alleviated the NGPV-induced changes, including shortened ileal villus length, increased crypt depth, and a significantly reduced villus-to-crypt (V/C) ratio. Furthermore, no significant differences were observed between the FMT-Y group and the PC group (Figure 5(b)). Subsequently, PAS staining was used to stain the glycoproteins in the intestinal mucosal layer into purplish red. The results demonstrated that NGPV infection resulted in a decreased intensity of PAS staining in the ileal mucosal layer of both the PC group and the FMT-Y group, whereas the FMT-A group exhibited a staining pattern comparable to that of the NC group. These findings suggest that NGPV infection induces damage to the ileal mucosal layer, and FMT-A treatment mitigates this effect (Figure 5(c)). Next, we observed that the Zona Occludens 1 (ZO-1) positive protein expression in the ileum was significantly reduced in both the PC group and the FMT-Y group, whereas ducks treated with FMT-A exhibited ZO-1 expression levels that were more comparable to those in the NC group (Figure 5(d,e)). Furthermore, the detection results of relative mRNA expression levels for the tight junction proteins (TJPs) Claudin-1, Occludin, and ZO-1 revealed that FMT-A treatment could enhance the mRNA levels of these 3 TJPs in the ileum. In contrast, the expression levels decreased in the FMT-Y and the PC groups (Figure 5(f)). Therefore, it can be concluded that FMT-A treatment effectively alleviates the reduction in intestinal TJPs expression induced by NGPV infection and protects the integrity of the intestinal barrier

### Adult duck fecal microbiota transplantation alleviates tibial dysplasia in SBDS ducks

**Figure 6.**
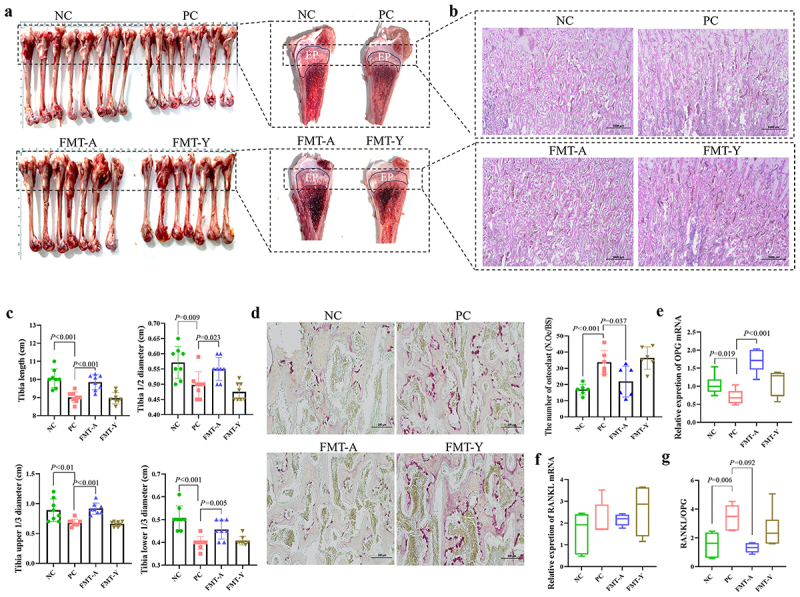
The impact of fecal microbiota transplantation from adult ducks on the tibia development of SBDS ducklings. (a): The tibias of the ducklings in each group were anatomically examined, and the proximal epiphyseal region of the tibia was analyzed through sagittal longitudinal sections. (b): The histological structures of the tibial proximal epiphyseal region in each group were compared using HE staining. (c): The differences in tibial bone dimensions among each group were compared, including tibia length, tibia 1/2 diameter, tibia upper 1/3 diameter, and tibia lower 1/3 diameter. (d): The number of osteoclasts per bone surface (N.Oc/BS) in each group was compared through TRAP staining. Compare the relative expression levels of OPG (e) and RANKL (f) mRNA in the tibia of each group (qPCR) and the ratio of RANKL/OPG (g).

The tibial tissue structure in each group of ducklings was compared, as illustrated in Figure 6(a). The sagittal plane of the tibia was longitudinally sectioned, revealing that the tibial epiphyseal (EP) area decreased in ducklings from both PC and FMT-Y groups, while the EP area in the FMT-A group remained similar to that of the NC group. Additionally, the trabecular bone in this region exhibited sparsity and thinning (Figure 6(b)). Subsequently, measurements were taken for the tibial length, upper 1/3 width, middle 1/2 width, and lower 1/3 width across all groups. The results demonstrated that NGPV infection caused a reduction in various dimensional indicators of the tibia in the PC and FMT-Y groups, while these effects can be mitigated by FMT-A treatment (Figure 6(c)).

To investigate the impact of NGPV infection on bone metabolism, TRAP staining was employed to observe positive osteoclasts. The results indicated a significant increase in N.Oc/BS due to NGPV infection, with the FMT-A group showing significantly fewer osteoclasts compared to the PC group (Figure 6(d)). Next, the relative mRNA expression levels of OPG (Figure 6(e)) and RANKL (Figure 6(f)) in the tibia were quantified. The results demonstrated that NGPV infection led to an upregulation of RANKL expression, a downregulation of OPG expression, and an increased RANKL/OPG ratio. In contrast, FMT-A treatment effectively reduced the RANKL/OPG ratio (Figure 6(g)). In conclusion, the fecal microbiota transplantation of adult ducks can mitigate the imbalance of bone metabolism and reduce bone mass loss in SBDS ducklings.

### Adult duck fecal microbiota transplantation suppresses the Th17 cells differentiation in NGPV-infected ducklings

**Figure 7.**
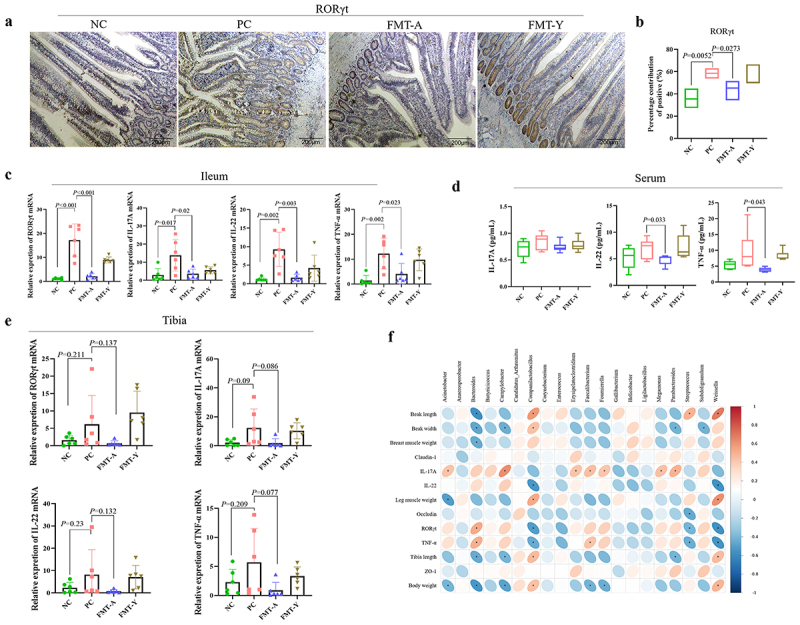
The impact of fecal microbiota transplantation from adult ducks on the Th17 cells in the ileum of ducklings. (a): Observe the protein expression levels of RORγt in the ileum of 4 groups through immunohistochemical staining. (b): The contribution of positive area in immunohistochemistry. (c): Compare the relative expression levels of RORγt, IL-17A, IL-22, and TNF-α mRNA in the ileum of each group using qPCR. (d): Compare the protein expression levels of IL-17A, IL-22, and TNF-α in the serum of each group using ELISA. (e): Compare the relative expression levels of RORγt, IL-17A, IL-22, and TNF-α mRNA in the tibia of each group using qPCR. (f): Heatmap of Spearman’s correlations between the differential ileum microbiota and growth performance, intestinal TJPs mRNA, and Th17 cells-related factors. The colors range from blue (negative correlation) to red (positive correlation). *p < 0.05.

To investigate the impact of FMT-A on Th17 cells in ducklings, in this study, IHC staining was performed on the ileum tissue to detect the protein expression level of retinoic acid receptor-related orphan receptor γt (RORγt), a specific transcription factor for Th17 cells. The results showed that RORγt was significantly expressed in the ileum tissue of both the PC and FMT-Y groups, whereas the expression in the NC and FMT-A groups was significantly decreased (Figure 7(a,b)). The relative expression levels of RORγt, IL-17, IL-22, and TNF-α mRNA in the ileum were quantified by qPCR. The results showed that NGPV infection significantly upregulated the mRNA levels of 4 indicators, while these in the FMT-A group were effectively decreased (Figure 7(c)). Subsequently, ELISA was employed to measure the protein concentrations of IL-17A, IL-22, and TNF-α in serum. The findings revealed that NGPV infection induced an increase in the concentrations of IL-17A, IL-22, and TNF-α (*p* > 0.05), while FMT-A treatment significantly decreased the serum levels of IL-22 and TNF-α (Figure 7(d)). Finally, the relative mRNA expression levels of RORγt, IL-17, IL-22, and TNF-α in the tibia were assessed. The results indicated that NGPV infection variably increased these markers in the tibia, and FMT-A treatment tended to reduce their mRNA expression levels (*p* > 0.05) (Figure 7(e)). In conclusion, adult duck fecal microbiota transplantation plays a crucial role in stabilizing the differentiation of Th17 cells in SBDS ducklings, thereby reducing the release of inflammatory cytokines.

The relationship among intestinal flora, duckling growth performance, mRNA levels of ileal tight junction proteins, and Th17 cell-related factors was investigated using Spearman correlation analysis. The results indicated that *Bacteroides*, *Campylobacter*, *Parabacteroides*, and *Subdoligranulum* were negatively correlated with duckling growth performance and positively associated with Th17 cell-related factors. Conversely, the abundance of *Companilactobacillus* and *Weissella* bacteria exhibited significant positive correlations with duckling growth performance and negative correlations with Th17 cell-related factors. Additionally, the expression level of Occludin was negatively correlated with the abundance of *Streptococcus* (Figure 7(f)). This means that bacteria that show a positive correlation with the Th17 cell-related factor may lead to exacerbated intestinal inflammation; conversely, those bacteria with a negative correlation may play a role in protecting the body during NGPV infection.

## Discussion

Clinical reports of SBDS predominantly occur in young ducks; this study further confirmed that ducklings are susceptible to NGPV while adult ducks are not. Furthermore, in our study, ducklings exhibited typical symptoms of SBDS, including weight loss, exposed tongue, and shortened tibia. These symptoms were consistent with the clinical, macroscopic, and histological changes in naturally infected ducks and ducks infected with NGPV reported by Chen [22]. This study also observed that the decreased muscle mass and abnormal bone development in the diseased ducks are also important factors leading to the low slaughter rate of the diseased ducks and causing economic losses for farmers. In addition, this study investigated the differences in fecal microbial composition between adult ducks and ducklings, and adult ducks exhibited greater abundance and diversity of fecal microbiota compared to ducklings. The microbiota structure in adult animals exhibits greater diversity compared to that in young animals. This pattern, observed in our study of ducks, is consistent across various species, including goats, chickens, and pigs, where fecal microbial diversity in young animals is significantly lower than in adults [23–25]. Next, we further analyzed the microbial composition of feces in ducklings and adult ducks. At the gene level, our results revealed that the abundances of *Romboutsia* and *Candidatus_Arthromitus* were significantly higher in the feces of adult ducks. *Romboutsia* has been shown to enhance the immune response in broilers and improve the balance of the intestinal microbiota [26]. *Candidatus_Arthromitus*, a segmented filamentous bacterium, plays an important but not fully understood role in poultry digestive health. Evidence from Danzeisen [27] indicates that the binding of these bacteria to the intestinal epithelium may promote early digestive development and health. Furthermore, NGPV infection caused substantial alterations in the gut microbiota structure of ducklings, while exerting only minimal effects on adult ducks. A comparable observation was made by Zhang [25] in pigs infected with porcine delta coronavirus (PDCoV). Based on these findings, we propose that modulating the intestinal microbiota and maintaining gut health could potentially reduce the susceptibility of ducklings to NGPV.

The FMT technology is currently receiving more and more attention from researchers. It has been confirmed to be an effective therapeutic approach for recurrent Clostridium difficile infection [28]. Furthermore, this technology has also been applied to the treatment of other intestinal diseases, including Crohn’s disease [29] and colitis [30]. Studies have demonstrated that puppies treated with FMT exhibit a more rapid resolution of diarrhea following canine parvovirus infection [31]. Additionally, FMT has been proven effective in reducing the susceptibility of pigs to PDCoV infection by promoting intestinal health and modulating the gut microbiota community [25]. In this study, FMT alleviated the symptoms of SBDS in ducklings and improved the growth performance of infected ducks. Moreover, our findings demonstrated that FMT can restore intestinal flora imbalance in infected ducklings. Specifically, FMT increased the *Firmicutes* abundance, decreased the *Bacteroidota* abundance, and significantly enhanced the F/B ratio in infected ducklings. The F/B ratio is widely recognized as playing an important role in maintaining normal intestinal homeostasis, and a decreased F/B ratio is frequently associated with inflammatory bowel disease [32]. Therefore, we used FMT to modulate the gut microbiota structure of ducklings, which is the reason for alleviating intestinal inflammation.

The intestinal mucosal barrier plays a critical role in nutrient absorption and waste elimination, while also serving as a robust defense mechanism against pathogen intrusion [33]. Consequently, enhancing intestinal development is vital for preserving organismal health and minimizing disease occurrence [34]. Villus length and crypt depth indicate the level of maturity and differentiation within the small intestinal mucosa [35]. As such, the V/C ratio is regarded as a key parameter for assessing the mechanical barrier function of the small intestine [36]. This study found that NGPV infection led to shorter ileal villi, increased crypt depth, and a decreased V/C ratio, while treatment with FMT-A could significantly increase the V/C ratio. Furthermore, the mucus barrier is the main defense layer that separates the microbiota from epithelial cells. It is formed by mucus secreted from goblet cells and coats the intestinal epithelial layer [37]. In this study, PAS staining was used to evaluate the ability of goblet cells and their secreted glycoproteins. FMT-A could increase the PAS-positive staining in the ileum of ducks infected with NGPV, suggesting that the colonization of fecal microorganisms in adult ducks can reduce intestinal mucosal barrier damage caused by viral infection. The intestinal epithelial barrier functions to separate the internal environment from harmful pathogens, thereby providing immune protection and maintaining homeostasis. Because intestinal epithelial cells are tightly sealed by TJPs, such as ZO-1, Claudin-1, and Occludin, intestinal microorganisms can modulate the permeability of the intestinal barrier by altering the expression and distribution of these TJPs [38]. Intestinal barrier damage can facilitate the translocation of microorganisms from the intestinal lumen to the subepithelial space, consequently triggering inflammatory responses [39]. Our study revealed that NGPV infection leads to intestinal microbiota disorders and intestinal barrier damage, which is consistent with the findings of Luo [40]. Moreover, they analyzed the correlation between intestinal barrier damage and intestinal microbiota disorders. In our study, the FMT was used to change the intestinal microbiota of ducklings. It was found that fecal microbiota transplantation from adult ducks could increase the levels of TJPs in the ileum of SBDS ducklings, confirming the importance of intestinal flora to the intestinal barrier of ducklings.

Osteoclasts are responsible for degrading the bone matrix and play a key role in the dynamic process of bone remodeling. Bone remodeling is a tightly regulated physiological process that involves complex interactions between osteoblasts and osteoclasts [41]. Specifically, the production of osteoclasts is facilitated by osteoblasts through the secretion of RANKL. RANKL binds to its receptor, RANK, located on the surface of osteoclast precursor cells, serving as a critical signal that promotes the differentiation of osteoclast precursors into mature osteoclasts [42]. Osteoblasts also produce OPG, which serves as a negative regulator of osteoclast activity. OPG competitively inhibits the binding of RANKL to RANK, thereby blocking signal transmission from osteoblasts to osteoclasts and suppressing the differentiation and maturation of osteoclasts [43]. Therefore, the ratio of RANKL to OPG can reflect the state of bone metabolism. In our research, we observed that NGPV infection resulted in an increased number of osteoclasts in the tibiae of ducklings and a higher RANKL/OPG ratio, which suggests an imbalance in bone metabolism and enhanced osteoclast-mediated bone resorption. This is a key factor contributing to the clinical susceptibility of SBDS ducks to fractures. Previous studies have demonstrated that intestinal microbiota significantly influence bone quality [44]. In our study, FMT from adult ducks can effectively mitigate abnormal bone growth in ducklings, reduce the number of osteoclasts, and decrease the RANKL/OPG ratio. These results confirm that modulating intestinal microbiota can alleviate bone developmental disorders in ducklings caused by NGPV infection. This result is consistent with the phenomenon observed by Zhang [45] using FMT technology in osteoporotic mice.

Since the disruption of the structure and distribution of intestinal microbiota is regarded as a potential characteristic of disease onset, and is closely associated with activation of Th17 cells [46]. The importance of the Th17/Treg cell balance in preserving intestinal immune homeostasis has been recognized. This is because Th17 cells are typically associated with autoimmunity and inflammation, while Treg cells function in an opposing manner to regulate these responses [47,48]. It has been reported that under pathological conditions, the intestinal flora can influence the immune system, particularly the differentiation of Th17 cells [49]. Our research also confirmed this point, which demonstrates that the differentiation of Th17 cells in SBDS ducklings can be alleviated by regulating the intestinal flora. Furthermore, reshaping the intestinal microbiota of chicks via FMT can modulate the balance between Th17 and Treg cells, thereby enhancing the growth performance in chickens [11]. This finding aligns with the approach of the present study. In the field of osteoimmunology, the imbalance of Th17/Treg and associated inflammatory factors has been demonstrated to play a critical role in bone metabolism dysregulation [50]. Studies have revealed that cytokines such as RORγt, IL-17A, and IL-21 expressed in the mucosa not only contribute to inflammatory bowel disease but also lead to elevated mRNA levels of IL-17A, IL-22, and TNF-α in knee joint tissues, which was observed in mouse models of rheumatoid arthritis [51]. In addition, there is evidence that Th17 cells can directly stimulate RNAKL expression and promote osteoclast maturation. On the contrary, reducing the interaction between Th17 cells and osteoclast precursors effectively inhibits osteoclastogenesis [13]. Our study demonstrates that NGPV infection induces overexpression of RORγt, IL-17A, IL-22, and TNF-α in the ileum, accompanied by increased expression levels in tibial tissue. These findings suggest that NGPV infection activates intestinal Th17 cells, influencing bone immune status and potentially contributing to bone metabolic imbalance.

Through correlation analysis, we found that there was a positive correlation between the abundance of certain bacteria (e.g. *Campylobacter*) and the expression of Th17-related cytokines. *Campylobacter* often colonizes in large numbers in the intestines of poultry and is a zoonotic bacterial threat [52]. The study has shown that the early colonization of *Campylobacter* in the cecum of chickens can lead to an inflammatory response [53]. This implies that in the model of our study, *Campylobacter* in the intestines of ducklings is likely to be one of the factors leading to the differentiation of intestinal Th17 cells caused by NGPV infection. Conversely, we also found that certain bacteria were negatively correlated with Th17-related cytokines, such as *Weissella*. Some studies have shown that *Weissella* can act as a probiotic to alleviate enteritis caused by *Escherichia coli* through competitive exclusion and regulation of the microbiota [54]. In this study, it means that *Weissella* may play beneficial roles in the intestines of ducklings infected with NGPV, such as maintaining the balance of the microbial community and alleviating inflammation. Of course, these are just speculations. Further research is needed to confirm the association between these bacteria and the differentiation of Th17 cells. And whether the increase in Th17 cells is directly linked to osteoclast maturation in duckling tibiae remains to be experimentally validated. In addition, in this study, the differentiation of Th17 cells was indirectly verified by detecting the levels of Th17-related cytokines and the expression of RORγt. This result has limitations. In subsequent studies, flow cytometry can be considered to directly observe the changes in the Th17 cell population.

In conclusion, this study confirmed that ducklings and adult ducks exhibit different susceptibilities to NGPV. The diversity of intestinal flora in adult ducks is higher compared to that in ducklings. Through FMT modification, it is possible to effectively restore intestinal microbial homeostasis in infected ducklings, mitigate intestinal barrier damage, thereby inhibiting Th17 cells differentiation, suppressing osteoclast maturation, and promoting the growth and development of NGPV-infected ducklings. These findings provide a theoretical basis for the development of novel strategies targeting gut microbiota modulation for the prevention and control of SBDS in young ducks.

## Supplementary Material

Supplemenary Information.docx

Graphical abstract.tif

## Data Availability

The 16S rRNA amplicon sequencing datasets generated in this study are available in the NCBI SRA repository, under BioProject numbers PRJNA1254916 and PRJNA1255372. NCBI link: https://www.ncbi.nlm.nih.gov/bioproject/PRJNA1254916/; https://www.ncbi.nlm.nih.gov/bioproject/PRJNA1255372/. The data that support the findings of this study are openly available in ScienceDB at: https://doi.org/10.57760/sciencedb.24181[55].
