# Supplementary material for: Adult duck fecal microbiota transplantation alleviates short beak and dwarfism syndrome in ducklings by inhibiting Th17 cell differentiation
Source: Virulence. 2025 Dec 16;17(1):2605745. doi: 10.1080/21505594.2025.2605745 (PMC12758264; doi:10.1080/21505594.2025.2605745)
Supplement: Supplemenary Information.docx [file KVIR_A_2605745_SM6939.docx]

Supplemenary Information for:

**Adult duck fecal microbiota transplantation alleviates short beak and dwarfism syndrome in ducklings by inhibiting Th17 cell differentiation**

Mandi Liu^123^, Weining Liu^12^, Kuan Zhao^12^, Wuchao Zhang^12^, Baishi Lei^12^, Yunhang Zhang^12^, Limin Li^1*^, Wanzhe Yuan^12*^

1 College of Veterinary Medicine, Hebei Agricultural University, Baoding, NO.2596 Lekai South Street, Hebei 071000, China.

2 National Research Center of Engineering and Technology for Veterinary Biologicals, Nanjing, 210014, China.

3 College of animal science and technology,Chongqing Three Gorges Vocational College. Chongqing, 404100, China.

* Corresponding author:

Limin Li

E-mail: [lilimin03@163.com](mailto:lilimin03@163.com)

Hebei Agricultural University, No.2596, Lekai South Street, Lianchi District, Baoding 071000, China.

Wanzhe Yuan

E-mail: [yuanwanzhe@126.com](mailto:yuanwanzhe@126.com)

Hebei Agricultural University. No 2596, Lekai South Street, Lianchi District, Baoding 071000, China.

**The supplementary information consists of:** Fig S1

Figure S1


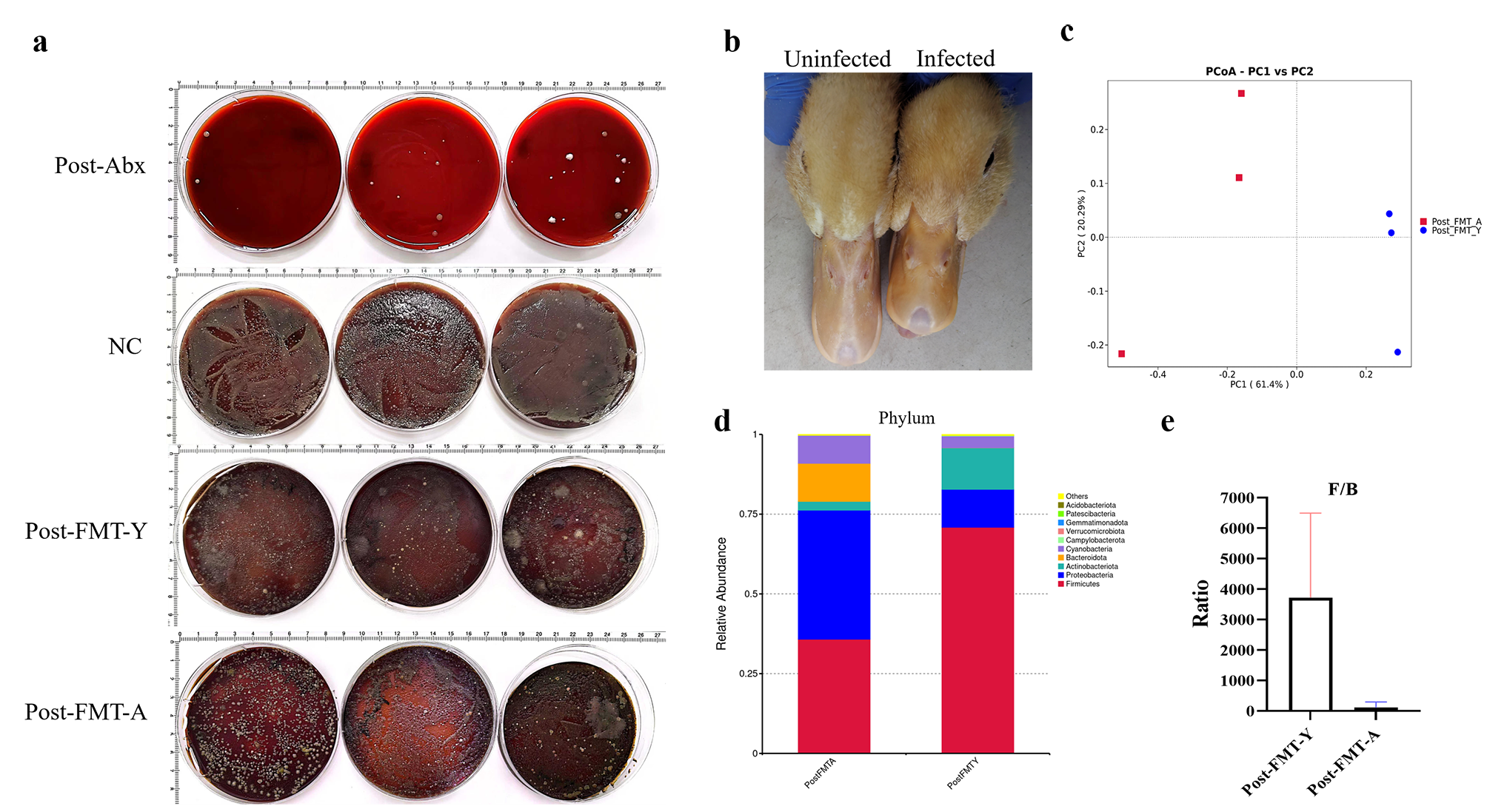


**Fig. S1 Confirm the successful modeling of intestinal microbiota depletion and reconstitution in the duckling model.** **a**: The bacterial colonies in the fecal samples were observed by coating blood plates with the feces of ducklings. **b**: Comparison between ducklings infected with NGPV (right) and uninfected ducklings (left). **c**: The β diversity of the fecal microbiota in the FMT-A and FMT-Y groups was compared at Post-FMT through PCoA. **d**: Composition of fecal microorganisms relative abundance at the Phylum level in the FMT-A and FMT-Y groups at Post-FMT through the column chart. **e**: The ratio of Firmicutes to Bacteroidetes (F/B) in the FMT-A and FMT-Y groups was compared at Post-FMT.
