# Supplementary figures and images for: Adult duck fecal microbiota transplantation alleviates short beak and dwarfism syndrome in ducklings by inhibiting Th17 cell differentiation
Source: Virulence. 2025 Dec 16;17(1):2605745. doi: 10.1080/21505594.2025.2605745 (PMC12758264; doi:10.1080/21505594.2025.2605745)

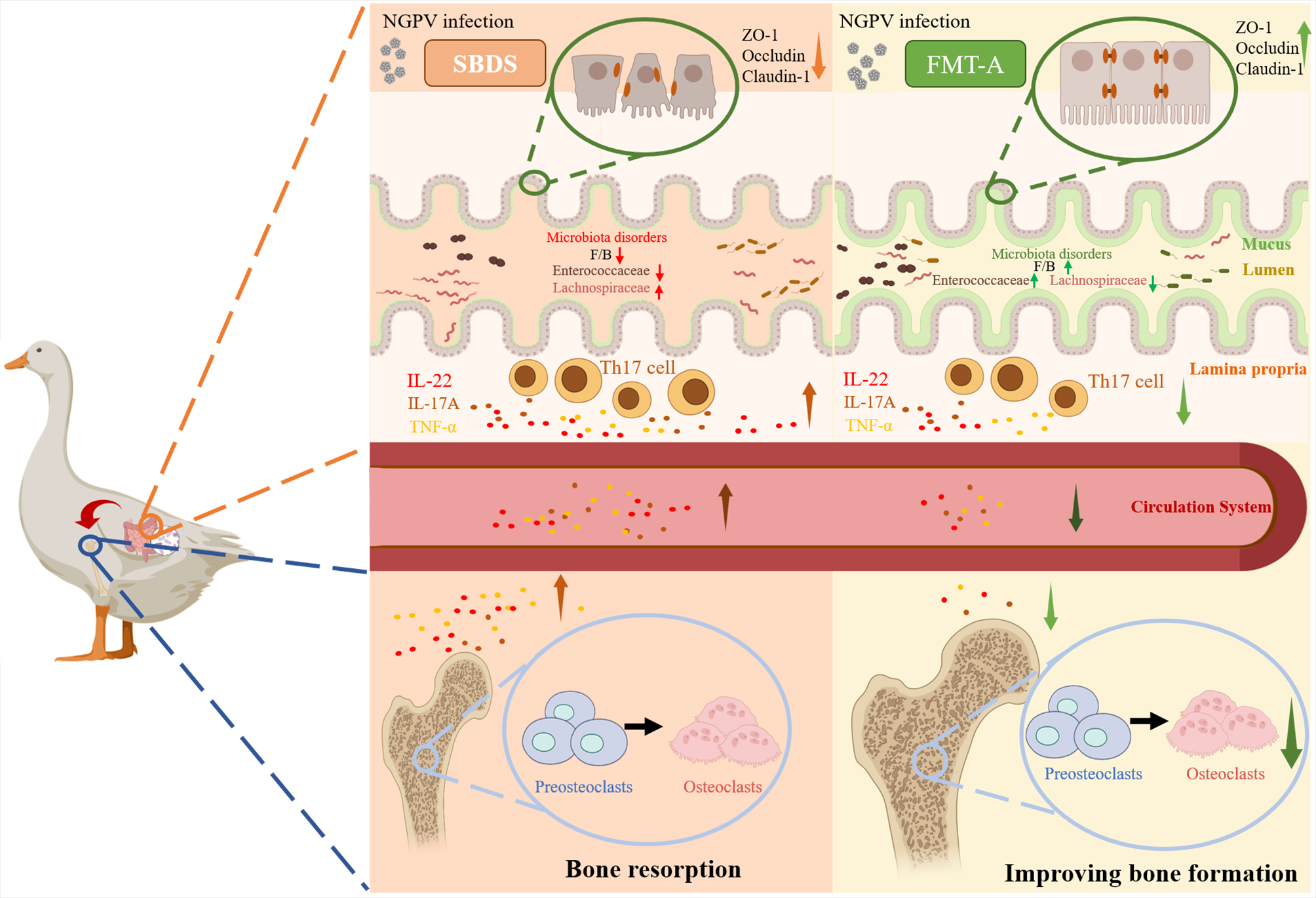

Supplement: Graphical abstract.tif [file KVIR_A_2605745_SM6938.tif]
